# Lea’s Series of Pocket Textbooks

**Published:** 1903-06

**Authors:** 


LEA'S SERIES OF POCKET TEXTBOOKS. Diseases of the Skin. A Manual for Students and Practitioners. By JOSEPH GRINDON, Ph. B., M. D. Professor of Clinical Dermatology and Syphilis, Washington University, St. Louis. Large duodecimo, pp. 377. Illustrated with 39 engravings. Series edited by BERN B. GALLAUDET, Demonstrator of Anatomy and Instructor in Surgery, College of Physicians and Surgeons, Columbia University, New York. New York and Philadelphia: Lea Brothers & Co. 1902.

In this manual Grindon has stated in a plain, terse manner the principal facts of dermatology. We miss, at the outset, the usual description of the anatomy and physiology, which in most textbooks serve to introduce the subject. This, however, may be just as well, since the student can easily obtain all such information from other works. This volume is described in the preface to be complete in its essentials for the use of students, and to be of real value to the physician as a means of refreshing his recollections and posting him to date. The subjects are discussed from a modern standpoint and with a praiseworthy freedom from hackneyed technicalities. The style is concise, the presentation of subjects is clear, and to each one is very properly assigned a special chapter.

The author takes rather singular liberties with recognised classification when he describes seborrhea under anomalies of secretion and pityriasis capitis under inflammation. The illustrations, which are reproductions by the author and Dr. John B. Kieber, are new and while some of them are very fine, others are simply execrable. In our opinion, the mounted chromolithograph, purporting to illustrate this premycosic stage of mycosis fungoids, is of doubtful utility, as it wholly fails to bring out the salient characteristics of the disease. One who should happen to pick up the volume at a bookstore, judging from its tawdry appearance, would probably conclude that he had chanced upon the latest flashy novel. G. W. W.

